# Molecular dynamics simulation reveals the possible druggable *hot-spots* of USP7

**DOI:** 10.18632/oncotarget.26136

**Published:** 2018-09-28

**Authors:** Mitul Srivastava, Charu Suri, Mrityunjay Singh, Rajani Mathur, Shailendra Asthana

**Affiliations:** ^1^ Drug Discovery Research Center (DDRC), Translational Health Science and Technology Institute (THSTI), Faridabad, Haryana, India; ^2^ Delhi Institute of Pharmaceutical Sciences and Research, Puspvihar, New Delhi, Delhi, India

**Keywords:** deubiquitinases enzyme, ubiquitin, docking, molecular dynamics simulation, free energy calculations

## Abstract

The plasticity in Ubiquitin Specific Proteases (USP7) inducing conformational changes at important areas has highlighted an intricate mechanism, by which USP7 is regulated. Given the importance of USP7 in oncogenic pathways and immune-oncology, identification of USP7 inhibitors has attracted considerable interest. Despite substantial efforts, the discovery of deubiquitinases (DUBs) inhibitors, knowledge of their binding site and understanding the possible mechanism of action has proven particularly challenging. We disclose the most likely binding site of P5091 (a potent USP7 inhibitor), which reveal a cryptic allosteric site through extensive computational studies in an inhibitor dependent and independent manner. Overall, these findings demonstrate the tractability and druggability of USP7. Through a series of molecular dynamics simulations and detailed quantitative analysis, a dynamically stable allosteric binding site near catalytic center of the inactive state of USP7 (site partially absent in active state), along with two newly identified sites have been revealed, which opens the avenue for rational structure-guided inhibitor designing in USP7 specific-manner.

## INTRODUCTION

Ubiquitin Specific Proteases 7 (USP7) is a member of ubiquitin specific proteases (USP) family that is extensively studied cysteine proteases in De-ubiquitinating enzymes (DUBs). Its interaction with wide range of proteins makes it an attractive therapeutic target due to its involvement in different oncogenic pathways as well as its role in metabolic, immune disorder and viral infections [1–3]. Its role in cancer progression [4] and in tumorigenesis has been well documented [5, 6]. USP7 deubiquitinates many proteins that are involved in cancer progression pathways such as p53, MDM2, BRCA1-A, p21 and Beta-catenin [6–10].

USP7 is an internally regulated protein having multi-domain architecture. The N-terminal has a TRAF-like domain, responsible for identifying substrate (like p53 and MDM2) [11–13] and at C-terminal it has 5 auxiliary domains termed as UBL12345 (Ubiquitin like domains). These auxiliary domains also interact with proteins like GMPS, DNMT1 [14] and ICP0 [15]. In between lies a catalytic domain (CD) of USP7 that performs its primary activity of cleaving ubiquitin from substrates [16]. The CD and UBLs are connected with a flexible 26 amino acid Connector helix (CH). In the inactive state of USP7, the residues of catalytic triad (C223, H464 and D481) are far apart therefore, it is unable to perform its protease activity [16], however, it gains active state upon ubiquitin binding [16]. The structural arrangement of USP7-CD revealed that the catalytic triad is inactive until the structural changes are induced by binding of ubiquitin. This internal regulation and changes in conformation of USP7 favors the possibility of identification of other possible inhibition sites. The knowledge of allosteric and other noncompetitive regulatory sites on USP7 have been of interest for the identification of its inhibitors. Indeed, the limited information about the possible druggable sites of USP7 appears to be the bottleneck for the identification of USP7 inhibitors.

The discovery of novel allosteric sites may offer an orthogonal mechanism for modulating the biological activity of USP7 and may provide improved selectivity to the inhibitors of USP7. Some low affinity (μM range) small molecule inhibitors like P5091, P22077 and HBX41108 have been reported for USP7. It is reported that treatment with P5091 or P22077 leads to MDM2 destabilization and p53 stabilization [8–14], resulting in tumor cell death *in-vivo* [15–17]. P5091 is able to overcome resistance to proteasome inhibition by bortezomib in multiple myeloma cells that overexpress USP7 [17]. Consequently, these compounds reduce medulloblastoma, colorectal and lung tumor growth in mice [13, 18] but it is unclear whether these effects are solely attributable to USP7 target modulation.

Recently, potent, novel, and selective inhibitors of USP7's have been developed using both rational and structure-guided design enabled by high-resolution co-crystallography [19–22]. Turnbull AP *et al*, Nature, 2017, have identified the most potent inhibitors at nanomolar range [19]. However, its role in trapping USP7 inactive, modulating its dynamics and dynamics associated modulation is still to explore. We have chosen inhibitor P5091 as a chemical probe as it is the most extensively studied potent (EC_50_ = 4.2 ± 0.9 μM), specific and selective inhibitor for USP7. It has shown inhibition activity both *in-vitro* and *in-vivo* studies [17]. Moreover, its inhibition mechanism and most likely binding site is not yet known. P5091 shows inhibitory activity in multiple myeloma, colorectal, ovarian and prostate cancers [17, 18, 23, 24]. The discovery of P5091 was done by using ubiquitin-phospholipase A_2_ enzyme (Ub-PLA_2_) reporter assay and a diversity-based library of small molecules was used for high-throughput screening [17]. Multiple crystal structures of USP7 are available, therefore, it is also more feasible to derive a large set of protein conformations using computational methods. This is driving structure-based drug design to move beyond static structures perspective to account for and embrace the flexibility of proteins. Since structural pharmacophore guide their ligand designing towards the formation of specific protein ligand interactions, a diverse set of protein conformations presents an opportunity to explore the chemical space more widely. Identification of most likely binding site on the surface of protein is a critical step for design of small molecules against a known target. The intricate mechanisms by which proteins are regulated often invoke binding at sites which can be viewed as allosteric sites separate from the catalytic site. Though USP7 is well studied, its dynamics with respect to whole protein, sub-domains, primary and secondary pockets dynamics is still unexplored at atomic level. To exploit the structure-dynamic-function and inhibitor's key information at molecular level, we have strategized a computational approach, which highlights possibility of identifying the binding site in USP7, and to best of our finding an allosteric binding site is revealed that looks promising for restraining USP7's biological activity. Moreover, this work open the ways for structure based drug discovery, which will eventually contribute in designing more potent inhibitors of USP7.

## RESULTS

### USP7 architecture into two states

The catalytic domain of USP7 constitutes a well characterized hand like architecture with finger, palm and thumb that is mainly responsible for its catalytic activity. The coordinates for catalytic domain were obtained from PDB-ID 4M5W [25]. From literature, we found that auxiliary domains are essential for biological function, therefore PDB-ID 5FWI [26] that contains CD, CH and also three additional domains as UBL123 was also used. Both the crystals have minor breaks in loop regions that were built and the optimum loop conformation was chosen on the basis of lowest energy. The structures obtained were energy minimized (*hereafter* M1 and M2) and used for further analysis. The rationale behind taking both the models in our study is to understand dynamic behavior of USP7-CD independently and in presence of other auxiliary domains.

### Exploring the druggable binding sites

**Scheme 1:**
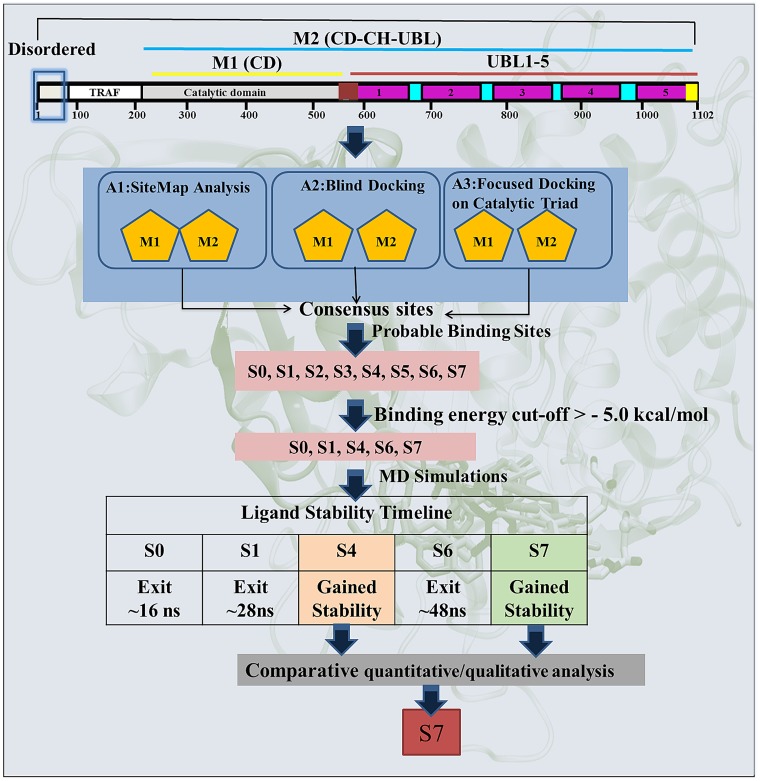
Schematic representation of the protocol used for the identification of most likely binding site and the binding mode of inhibitor P5091 on M1 and M2

**Figure 1:**
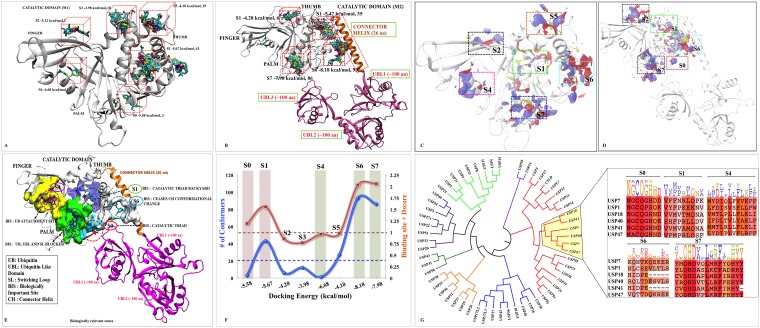
Possible binding sites and poses found by docking and SiteMap correlating with biological significance of each site M1 and M2 are shown as cartoon. The blind docking P5091 (rendered in licorice and atom wise, C:cyan, H:white, N:blue, O:red and S:yellow) distributions at (**A**) M1 and (**B**) M2. The SiteMap analysis on (**C**) M1 and (**D**) M2. (**E**) The possible consensus binding sites (rendered in surface view) with their respective biological importance. (**F**) Combined evaluation of all sites based on docking energy, site-score and Dscore with respect to number of conformation for each site. P5091 at different sites is notated with different color: S0-Red, S1-Green, S4-Magenta, S5-Orange, S6-Cyan and S7-Blue. (**G**) Conserveness through phylogenetic analysis of probable sites.

To identify the most likely (diverse pocket selection) ligand binding sites, ligand-dependent (blind docking) and ligand-independent (SiteMap of Schrodinger) strategies were used on M1 and M2 models (Scheme 1-Route A1 and A2 respectively). Blind docking (BD) on M1 revealed 6 different sites S0, S1, S2, S3, S4 and S5 (Figure 1A). BD on M2 also predicted consensus sites S2, S4, S5 and two new sites S6 and S7 (Figure 1B). Although, a small number of low energy conformers were observed at catalytic site, but given the biological significance of this site, an additional focused docking was performed (including catalytic triad residues C223, H464 and D481) (Scheme 1-Route A3). The low cutoff for selection of poses was taken (docking energy: −5.00 kcal/mol; and number of conformers: >20) in order to identify the maximum possible sites, and minimizes the false positives [27, 28]. From three different docking protocols the five main sites viz. S0, S1, S4, S6 and S7 were picked. The sites S2, S3 and S5 were removed after docking process, as they do not satisfy the minimum cutoff values. Adding more information to the heuristic search done earlier through BD, we also added a robust module to predict the possible binding sites in a ligand independent manner. We identified six binding sites S1, S2, S4, S5, S6 and S7 (Figure 1C and Supplementary Table 1) on M1 and 5 binding sites S0, S1, S2, S6 and S7 were identified on M2 (Figure 1D and Supplementary Table 2). Except site S4, from M1 and site S0 from M2, rest of the sites is common in both the models. Moreover, the site S3 was not identified in M2 and sites S2 and S5 do not reflect the potential druggability values by SiteMap, therefore, the rejection of these sites corroborates well with the BD outcomes. These results clearly indicate that S1, S6 and S7 have come up as consensus sites with better docking energy and druggability score (Dscore) (Supplementary Tables 1 and 2 respectively), hereafter, sites S1, S6 and S7 are selected for further analysis. Additionally, we also include S0 and S4 sites as an exception despite having their low cutoff values, because they localized at/near biologically key regions of USP7.

Hence, taking both the methods ligand-dependent and ligand-independent with reliable descriptors (docking energy, number of conformations, site score and Dscore), we funneled down five possible sites S0, S1, S4, S6 and S7 for more detailed analysis (Figure 1E and 1F).

### Correlation between identified sites and its biological relevance

In order to anticipate the mechanism of inhibition of the predicted sites it is imperative to understand the underlying biological relevance of all these sites. From literature we identified site S0 occupies the catalytic triad, seems to be the most potential site as it will directly inhibit the catalytic activity of USP7 [16]. The site S1 lies near to N-term (TRAF domain) which is responsible for substrate recognition [11–13]. Targeting this site might not allow USP7 to identify its substrate that will eventually inhibit its DUB's activity. The Site S4 occupies the ubiquitin-positioning site where inhibition at this site might not allow ubiquitin to get itself deployed into finger domain. It is reported that USP7 shifts its dynamic equilibrium from inactive to active state by positioning its C-terminal into catalytic domain [29]. The regulation of this functional activity is capped by CH and hence site S6 which is forming a bridge between CD and CH will provide important interactions that might curb USP7 to become active. The site S7, which possibly block attachment of ubiquitin C-term tail to the catalytic site and UBL C-term tail strongly interfere with ubiquitin binding seems another potential site as well.

Previous studies have reported that there is always an evolutionary pressure on the binding sites to maintain its conserveness for a biological function to happen [30, 31]. Therefore, we scrutinized chosen binding sites by performing a discrete phylogenetic analysis for the assessment of residues conservation. The rational in doing so is to judge our predicted binding sites by associating conserveness with the biological function. Through phylogeny we found USP1, USP18, USP40, USP41, USP47 sharing the closest similarity to USP7 and then a motif pattern was generated for every site (Figure 1G). The binding site residues of USP7 at respective sites were taken to form the motif pattern (Supplementary Table 3). We found the site S0 and S7 are highly conserved (Figure 1G). Undoubtedly site S0 has to be conserved as it forms catalytic site which provides the base for biological function to USP7. Conserveness at S7 has given subtle clues for allosteric inhibition and secondary binding site as it lies in the path of ubiquitin C-term tail and also near to catalytic center which might hinder USP7 biological function. No wonder other sites S1, S4 and S6 are less conserved between selected members of USPs as they might be responsible for different regulatory mechanisms. The S6 is the least conserved site as this site belongs to the proximity of CH, which is a unique element of USP7. From this data, we are speculating S7 as the most favored site. Probabilities are numerous and also this correlation justifies the importance of these sites and hence the MD simulations and free energy calculation was conducted.

### Characterization of binding sites on the basis of stability profiling

**Figure 2:**
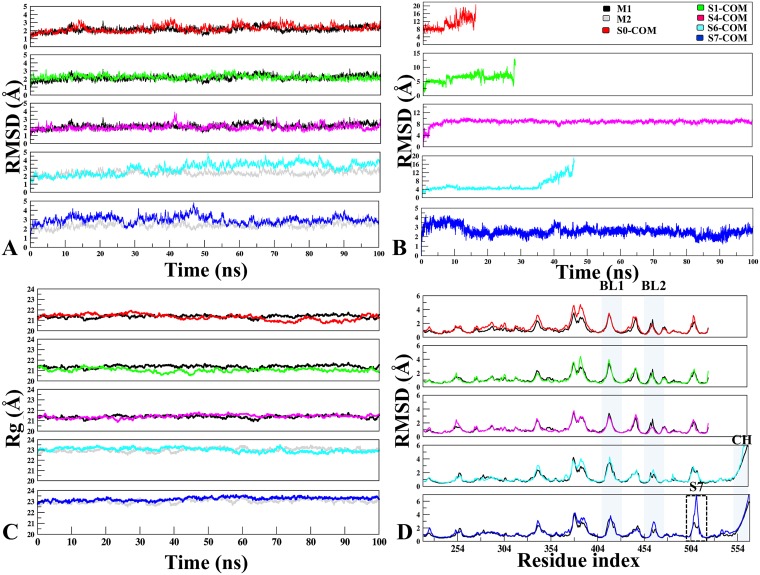
The monitoring of MD trajectories (**A**) APO-vs.-Docked Stability: Root Mean Square Deviation (RMSD) of Cα atoms for APO-vs.-Docked COMs at five different sites (Red: S0-COM, Green: S1-COM, Magenta: S4-COM, Cyan: S6-COM, Blue: S7-COM, respectively). (**B**) Ligand Stability: RMSD of P5091 at five different sites. (**C**) Radius of gyration. (**D**) Root Mean Square Fluctuation: Flexibility of M1 (in black) and M2 (in grey) in APO and COM forms, estimated from the RMSF values along the equilibrium MDs. The important structural areas in USP7 are notated as Blocking loop (BL1 and BL2), Binding site area of S7 and Connector Helix.

Multiple MD simulations were carried out to determine the stability of multiple USP7 and its complexes. Since our focus is to identify the potential sites, therefore, binding site characterization through dynamics is essential to map the druggability of each site with the evolution of time. The RMSD/RMSF analysis was performed to access the dynamic stability and fluctuations of the proteins. Conformational and geometrical properties were examined to understand the structural and dynamic changes induced by binding of P5091 at five different complex systems (S0, S1, S4, S6 and S7) with respect to APO. In all the systems (Figure 2A), there were no significant difference observed in APO and COM of whole protein, except sites S6 and S7. This deviation in S6 and S7 can be attributed to the proximity of highly flexible loops like CH, BL1, BL2 and SL.

### Unstable associations

The magnitude of fluctuations indicates that in all the systems P5091 attained stable conformations at initial 5 ns of simulations (Figure 2B). Furthermore, we noticed in S0 and S1 trajectories starts destabilizing and P5091 exits from these pockets after ~10 ns and ~30 ns, respectively. This finding is further reflected in thermodynamics data (Supplementary Figure 1). Most striking behavior was observed in case of S6, where P5091 despite attaining highest cutoffs, docking energy and good number of conformations, it moved out from the S6 site after ~45 ns of MD simulations. Therefore, it would be worth to explore the reason behind instability and escape of P5091 at S6 despite being the best site in the docking studies. It is also interesting to observe that low docking energy at site S0 and S1 is due to the weak interaction with P5091 that eventually leads to its escape.

### Escape of P5091 from S6 binding site

At static docking pose P5091 forms good interactions with residues Q219 and K277 of CD and E551 and R555 of CH where the residue pairs Q219@R555 and K277@E551 are acting as the terminal points involved in establishing the interaction bridge formed between CD and CH. Hence, taking L544@CH as an anchor residue of these bridges, we formed two triads T1 (L544-K277-E551) and T2 (L544-Q219-R555). Area of both the triads formed between CD and CH were used to study the conformational changes during the course of MD in APO and COM systems. During simulation time-scale the conformational changes result in a dramatic expansion in the area of both the triads in COM with respect to APO (Supplementary Figure 2). This increase in area is due to the high flexibility of CH, where the bending disruption occurs in lower half of CH (Supplementary Figure 2) while upper part was found rigid. The reason for flexibility of CH might be due to the charged residues that are residing at CH (K554, R555 and R558) and five lysine residues (K217, K272, K277, K278 and K281) of CD, out of which we have chosen K277 as our reference for area calculation. Though this method for area calculation is not an accurate protocol, but still it provides tentative information about the changes at S6 site. Laying down an assumption, we could say that these positive surfaces at both ends could repel CH away from CD. Thereby, P5091 doesn't prove to be potential at this site because as the degree of movement of CH increases, P5091 losses its contact and moved out from the binding site.

### Stable associations

Stable association was observed throughout 100ns of simulations at sites S4 and S7. A significant movement ~7.8 Å of P5091 was observed from its docked pose to stable MD pose (dock-to-MD transition) at S4 site of finger domain. This dock-to-MD pose transition could be the reason of its initial high RMSD value, though after attaining the MD pose the S4 is stable throughout the simulation (Figure 2B). At S7, the average RMSD is ~3.0 Å, indicating the MD trajectory of S7 attain the plateau with least changes in the trajectory. The Radius of gyration (Rg) was also calculated to confirm the compactness of COMs during the simulations (Figure 2C). All systems were compacted with 21–23 Å, which defines that in the APO and COM states, there is no significant deviation was observed and the compactness is maintained throughout the trajectory. From the RMSF, there were no significant fluctuations were found at peak representing BL1 and BL2 while comparing with different systems but overall their fluctuation remains high due to its innate flexible nature (Figure 2D). A significant fluctuation is observed near S7 binding site (500–510) which states that P5091 stability at this site has put its effect on surrounding residues. Lastly, zone-depicting CH whose fluctuation reaches to 7.0 Å is because of its flexible nature.

From the above analysis S7 appears as the most probable binding site for P5091. However, stable interaction was observed at S4 site, too. Hence, both the sites S4 and S7 are subjected to thermodynamics calculations pursued with comparative analysis of the most likely binding site.

### Residue-wise mapping of S4 and S7 Sites

**Figure 3:**
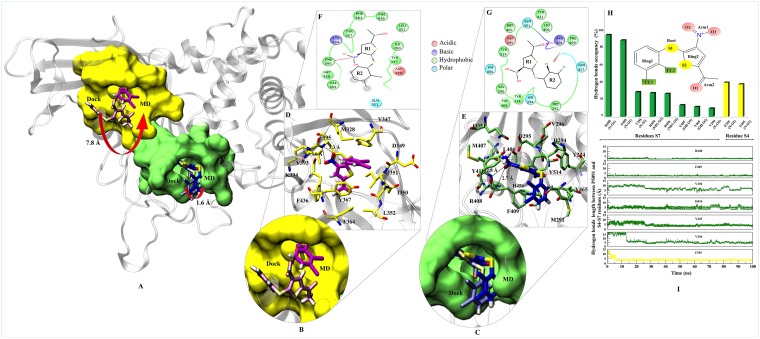
Binding modes and interaction map of P5091 at two most potential sites Shown conformations of P5091 are representatives MD frames featuring the lowest rmsd with respect to the average structure of complexes, as extracted from MD trajectories. Proteins are rendered in cartoon and P5091 is licorice in atom-wise mode, C: magenta (S4) and dark blue (S7), H:white, N:blue, O:red and S:yellow. (**A**) The molecular surface of residues lining the binding sites is highlighted at S4 (yellow) and S7 (green). Also the movement from Dock-to-MD most stable state is also highlighted from light-magenta to dark-magenta and light-blue to dark-blue at S4 and S7 sites, respectively. Close up view of P5091 at S4 (**B**) and S7 (**C**). Interaction maps of P5091 at S4 (**D**) and S7 (**E**). The ligand-wise 2D interactions map of P5091 at S4 (**F**) and S7 (**G**). (**H**) The H-bonds occupancy throughout trajectory for S4 (in yellow bar) and S7 (in green bar). (**I**) The occupancy is defined as the percentage of simulation time in which a specific hydrogen bond exists.

The interaction map of complexes at S4 and S7 sites were determined to identify the binding mode of P5091 and the key residues, by taking the lowest RMSD frame from the average structure extracted from the MD trajectories, respectively. To achieve the most stable state a significant movement (~7.8 Å from the center of mass of docked-vs.-MD pose) of P5091 was observed at S4. While in case of S7 a slight movement (~1.6 Å) was observed (Figure 3A–3C). The interaction map has shown that residues lining the binding pocket at S4 are mostly hydrophobic in nature (M328, Y347, I350, L352, F364, Y367, V393, F395 and F436), one acidic (D349), one polar (Q351) and one basic residues (K394) constitute the pocket (Figure 3D and 3E). At S7 the binding site residues are also hydrophobic (Y224, M292, V296, L406, M407, F409, Y411, Y465 and Y514) in nature, four polar residues (H294, Q351, Q417 and H456), one acidic (D295) and, basic (R408) residues that constitutes this site (Figure 3F and 3G). At S4, residue F395 is involved in forming hydrogen (H) bonds with two atoms of P5091 i.e. F395:N@P5091:O2 (3.3 Å) and F395:N@P5091:O3 (3.0 Å) which remain stable throughout the timeline of MD simulation (Figure 3I) while at S7 two residues are mainly establishing H-bonds, R408:N@P5091:O1 (2.8 Å) and F409:N@P5091:O1 (2.7 Å) (Figure 3H). These two residues also display constant distance throughout the simulation timeline (Figure 3I). Interestingly, an aromatic box of residues is observed at S7, which includes residues Y224, F409, H456, Y465 and Y514 contributed substantially. The rotameric analysis was performed on residues R408 and F409 in APO/COM, and we observed substantial conformational change in residue R408, especially at χ^1^ and χ^4^ angles (Supplementary Figure 3). This diverse state of residues indicating the different states of binding site residues in APO/COM possibly through induced fit mechanism.

### Binding free energy calculations

After understanding the dynamic properties of P5091 at all different sites, we calculatedthe free energy of binding (*Δ*G_bind_) [32–34]. The results indicate that P5091 binds more favorably at S7 (*Δ*G_bind =_ −34.23 kcal/mol) than S4 (*Δ*G_bind =_ −19.84 kcal/mol) (Supplementary Figure 4). Further analysis of binding energy components reveals that binding to both sites S7 and S4 is governed by mainly non-polar interactions as evident from high van der Waals (vdW) contributions and favorable non-polar solvation energies. The vdW contribution is -39.76 kcal/mol at S7 while it is −24.58 kcal/mol at S4 site (Supplementary Figure 4). The electrostatic contribution is also higher at S7, with the value of −11.42 kcal/mol, than S4 with value of −5.61 kcal/mol. Also the polar solvation penalty is more at S4 (12.11 kcal/mol) as compared to S7 (20.22 kcal/mol)(Supplementary Figure 4).

### Identification of binding site ‘*hot-spot*’ amino acids

**Figure 4:**
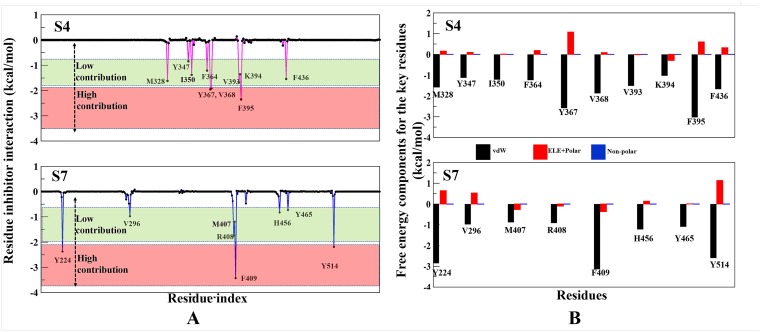
Contributions of the binding free energy components of P5091 at S4 and S7 (**A**) Per-residue decomposition of the binding free energy at S4 and S7 sites based on thermodynamic calculations on all residues. (**B**) The quantitative per-residue decomposition of binding free energy into contributions from the van der Waals (vdW), electrostatic, polar, and non-polar solvation energy for identifying key residues (from panel **A**) of both systems S4 and S7.

**Figure 5:**
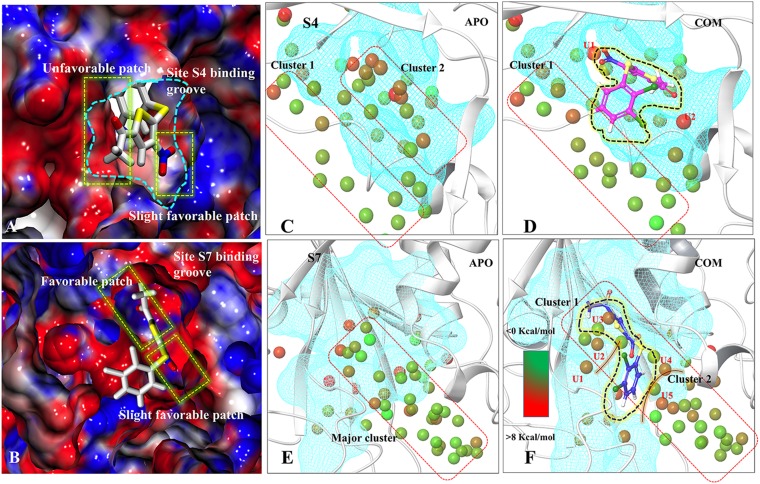
Characterization of binding sites via electrostatic potential calculations and water-map analysis Panels (**A**) and (**B**) are showing the electrostatic surface view of S4 and S7 sites. The positive and negative electrostatic potential are highlighted in blue and red, respectively. Hydration sites identification at S4-APO (**C**) and S4-COM (**D**), S7-APO (**E**) and (**F**) S7-COM. The unstable waters in COM which are named as U1, U2, U3, U4, U5. Color gradation of the hydration sites is based on free energy relative bulk water: Favorable/stable waters are shown in green and unfavorable/unstable waters are shown in red.

The per-residue binding free energy decomposition was performed to identify key inhibitor-residue pairs (Figure 4A). At site S4, residues M328, Y347, I350, F364, Y367, V368, V393, K394, F395 and F436 with binding energy in the range of −0.5 to −2.5 kcal/mol were identified as ‘*hot-spot*’. Amongst all residues, F395 has shown the highest binding energy contribution. Similarly, at site S7, residues Y224, V296, M407, R408, F409, H456, Y465 and Y514 were identified with binding energy in the range of −0.5 to −3.5 kcal/mol, while residue F409 has shown the highest binding energy. Though the number of interacting residues are almost same, a high binding energy contribution by each residue was higher in S7 than S4 (Figure 4A). For site S4 only residue F395 has shown higher energy contribution (−2.4 kcal/mol), while in S7, three residues Y224, F409 and Y514 have shown higher energy contribution (−2.2 kcal/mol, −3.5 kcal/mol and −1.9 kcal/mol), respectively. These residue-wise high binding affinity contributions corroborate nicely with H-bonds outcomes, as these residues are involved in established consistent H-bonds with P5091. Also, the per-residues binding free energy of all *hot-spots* was decomposed into contributions of vdW, the sum of electrostatic term in the gas phase and polar solvation energy and non-polar solvation energy for the above eight residues in both complexes (Figure 4B). It is worth observing that the dominant favorable interactions come from vdW interaction in both the systems with the energies in the range of −1.0 to −3.0 kcal/mol for the eight residues and other two terms contribute slightly to the binding free energy. At S7 residues Y224, F409 and Y514 are providing vdW interaction upto ~ (−3.0 kcal/mol) but only residue Y367 and F395 are showing higher vdWs interactions in site S4. In S4 only favorable electrostatic contribution is made by K394, while in S7 M407, R408 and F409 makes favorable electrostatic contributions. The S4 site offers a major negative potential to O1 atom of P5091 by residues D349 and E371 that challenges its complementarity while residue K394 is offering slight favorable patch to its O3 atom (Figure 5A). At S7, two basic patches are providing favorable interaction to P5091. Atoms O1 and O3 of P5091 are complemented by R408 and H456, respectively (Figure 5A and 5B). This result supports that S7 is more favorable site than S4. Hence, from the free energy analysis we also concluded that the electrostatic architecture of S7 is much better than S4.

### Water dynamics analysis

The role of water in ligand binding and its application in structure-based drug design is well documented [35]. In complex formation, the presence of water molecules can contribute to binding affinity by bridging protein-ligand interactions or by being displaced upon complex formation [36]. These phenomena are challenging to demonstrate at molecule level. We used WaterMap to study the dynamic behavior of water in COMs (S4 and S7) with respect to APO. WaterMap algorithm helps to determine the efficiency of ligand to displace the water molecules from receptor binding site so that it may get better packing into the pocket. At S4 (APO-vs-COM), it was observed that, two main water clusters, cluster1 and cluster2 are formed in APO. In case of COM, P5091 displaces the water molecule from the cluster2, however, the water molecules of cluster1 remain the same (Figure 5C and 5D). In the case of S7, only one major cluster is found in APO. In COM, the major cluster is segregated into two minor clusters by displacing the water molecules (Figure 5E and 5F). Moreover, the efficiency by which ligand displaces water from binding can be directly correlated with its potential to bind at that site. Seven hydration sites were displaced by P5091 when it binds at S4, however, nineteen sites were displaced at S7 site. The displacement of water significantly contributes in the space formation that was utilized by P5091 for its own packing. These water analysis values also support the better druggability of S7 than S4. Additionally, we also identify five water molecules (U1, U2, U3, U4, and U5) at S7 which forms unfavorable interactions. The information of unfavorable water interaction is vital and can be used for designing more potent derivatives of P5091.

### Dynamical changes observed in the key biological regions of USP7: possible mechanism of inhibition

To assess the impact of P5091 binding on USP7 at S4 and S7 sites, the key regions (SL, BL1, BL2, S7 binding area and the finger domain/ubiquitin attachment site) were picked and compared the conformational changes in APO and COM (Supplementary Figure 5A). All these regions are deemed to be important for biological function of USP7 as they are either involved in the recruitment of ubiquitin and/or involved in the proteolytic activity [16]. BL1 and BL2 provides the floor to ubiquitin tail that extends to catalytic center [16]. SL, an allosteric regulator is known to be important for the specificity of compounds [20]. The finger domain provides a perfect shell for ubiquitin to fit its globular architecture into it, and S7 binding area which was chosen in order to see any significant changes upon P5091 binding. From RMSD analysis, the dynamic comparison between S4 and S7 suggests that the key regions are affected when P5091 binds at S7 (Supplementary Figure 5B). At site S7, other than SL, the significant conformational changes were observed in all the selected regions. Residues of BL1, BL2 and binding site region, shows a considerable conformational deviation in COM state rather than APO. The opposite trend of RMSD was observed at finger sub-domain (includes S4 pocket residues also), in which the two fold increased RMSD was observed in APO than COM (Supplementary Figure 5B4). The dramatic conformational change induced by P5091 at finger domain (and since finger domain is being portrayed as shell for ubiquitin), this conformational instability might not allow ubiquitin to dock at this region.

Thus, this data also confirmed S7 to be more pronounced site for P5091 therefore more dynamic characterization was performed at S7.

### Breathing motion analysis of S7 through binding site's volume analysis

**Figure 6:**
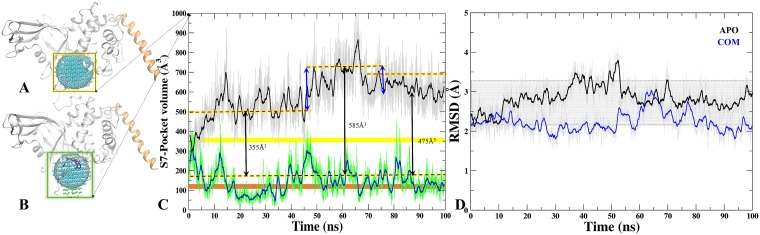
Tracking the breathing motion of site S7 through volume analysis The grid-points of inclusion sphere encompassing binding site S7 for APO (**A**) and COM (**B**) displayed in blue and in green. (**C**) Volumes throughout 100 ns trajectory of site S7 for APO (black) and COM (blue), Static value of volume for X-Ray-APO and APO’ is shown in horizontal yellow and orange bars, respectively. (**D**) The RMSD graph of binding site residues of S7 for APO (black) and COM (blue) throughout MD time scale.

We calculated the volume of all sites predicted on M1 and M2 through SiteMap (Supplementary Tables 1 and 2). Moreover, to understand the binding site dynamics of S7 in APO/COM, we performed the volume analysis throughout the trajectory (Figure 6A and 6B). The significant differences were observed in the volume of APO and COM trajectories which indicates its breathing motion (expansion and reduction) (Figure 6C). In APO, at first half of the MD simulations, the average volume of the site is around 550 Å^3^ while in the next half, the average volume increased to 700 Å^3^, however, the maximum volume was 900 Å^3^ observed at 65ns (Figure 6C). In case of COM, by contrast the volume remains consistent at 300 Å^3^ throughout the trajectory (Figure 6C). Indeed, in last 30 ns the volume of site considerably reduced to 165 Å^3^. Furthermore, the volume was also compared with APO’ (ubiquitin bounded crystal) and we noticed that in COM the volume drops below the APO’ state (Figure 6C), indicating a frozen state of S7 site in the presence of P5091. These values reflect clearly that S7 is dynamic and flexible site in APO and APO’ and the binding of P5091 reduces its flexibility thereby increasing the compactness of this site significantly (Figure 6D).

### Dynamics insight to site S7 and its comparison with published experimental data

**Figure 7:**
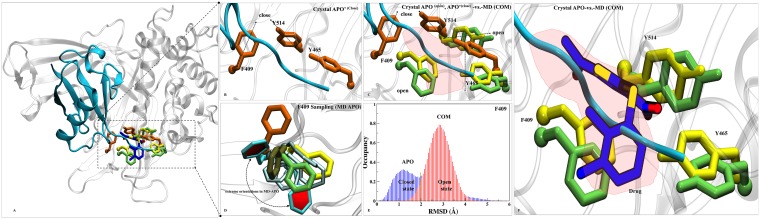
Dynamics insights at site S7 (**A**) Overall structure of APO’ PDB-ID: 5JTJ, represented in cartoon representation. (**B**) The ubiquitin bounded state (APO’) describes closed conformations of residues F409, Y465 and Y514 (orange in color and rendered in licorice). (**C**) Superimposition of crystals: APO’ (orange), APO (yellow, PDB-ID: 4M5W) and MD-COM (lime) states has shown in open and close states of F409, Y465 and Y514. A red patch is highlighted which pacifies the available space at S7 for P5091. (**D**) F409 sampling in MD-APO (cyan) observed after overlay with APO, APO’, MD-COM and Co-crystal (tan, PDB-ID: 5NGE). The distributions of F409 are shown in Cyan (two extreme positions (in dark-cyan) and its transient states (light-cyan). (**E**) The histogram of F409 has shown the existence of bi-model distribution in APO ((open/close, blue) than COM (close, red). (**F**) The space created by this conformational sampling is occupied by P5091 (blue atom wise: licorice) speculating blocking of ubiquitin c-term tail (in cyan).

From all the analysis, S7 turns out to be the most likely binding site of P5091 which is localized at thumb-palm cleft, that guides the ubiquitin C terminus into the catalytic site (C223, H464, D481). Thus a detailed insight conformational investigation was required to highlight the dynamic importance of this site. From the trajectory analysis, we found three *hot-spot* aromatic residues (F409, Y465 and Y514) that are contributing significantly in interaction with P5091, and also undergoes conformational sampling into two different states i.e from open (APO) to closed (APO’) states. To understand the conformational changes, we superimposed five states of USP7: a) APO’ i.e USP7-ubiquitin bounded state (PDB-ID: 5JTJ), b) APO i.e USP7-without ubiquitin state (PDB-ID: 4M5W), c) MD-APO state, d) MD-COM state and e) Co-crystal (PDB-ID: 5NGE) (Figure 7A). In ubiquitin-bounded crystal, these residues forms closed state and functions as a gatekeeper residue (thumb domain) for positioning of C-terminal of ubiquitin into catalytic site (Figure 7B). The proper positioning of the ubiquitin C-terminal into catalytic site requires significant conformational changes in certain residues to form the tunnel [20]. These residues are involved in tunnel formation by establishing the intra H-bonds (between Y465 and Y514) and facilitate the entrance of ubiquitin's C-term tail. However, in APO (open state) these three residues are falling apart (Figure 7C). This observation nicely testified from the APO-MD data where we sampled different rotameric states of F409 (Figure 7D) and Y514 (Supplementary Figure 6A). Binding of ubiquitin into finger domain of USP7 induces conformational changes by which it locks the residues into close state and able to place its C-term into catalytic site. From APO-MD, the occurrence of close state of key residues (equivalent to crystal) is ~23%, while the occurrence of open state is ~39% and many intermediate states are also observed (Figure 7D). The binomial distribution through APO-MD simulation of F409 (Figure 7E) and Y514 (Supplementary Figure 6B) clearly indicates the existence of these residues in two different forms. The identification of more expanded open state is the rare finding and was not found in any crystal data. It seems that in open state, the intra H-bonds between Y465 and Y514 move apart due to conformational changes, produced the extended binding site. We observed a concerted movement between F409 and Y514 as when F409 is at fully open state (solvent exposed), Y514 at intermediate state and vice-versa. This holds true in MD-complex state too, as all three residues can be seen in open state only (Figure 7C). The increased volume size of S7 site in APO state is targeted by P5091. The binding of P5091 in this state possibly block USP7 into auto-inhibited state by reducing the elasticity of the pocket residues drastically. The residue Y465 is forming H-bonds with O2 and O3 atom of P5091 and on the other side Y514 lies horizontally while a very stable association is formed between F409 and P5091. The tight packing of P5091 with F409 will not allow ubiquitin to extend its tail to catalytic center. Hence, P5091 acquired this space and perform its inhibition mechanism where it blocks the ubiquitin C-term tail (Figure 7F). The switching loop conformation in APO possibly prevents Y465 thumb interaction. The unrestrained conformation of Y465 allows nearby Y514 to sit flat in the ubiquitin-binding channel (Figure 7C), and in contrast to other USPs, this residue does not interact with the switching loop. Both Tyr residues conformations create space for P5091 binding. Together, the positions of Y465 and Y514 in the ubiquitin-binding channel, enabled by a unique conformation of the switching loop in APO, allow P5091 to specifically target USP7.

The binding location of S7 matched very nicely with recently published co-crystal PDB-ID: 5NGE (8WK) [19]. We superimposed our best stable state of COM over 5NGE and we observed a high similarity between interaction patterns of both the molecules which strengthen to our findings substantially. Three residues (Q297, F409 and Y465) are forming H-bonds with inhibitor 8WK, while two residues, R408 and F409 are forming H-bonds with P5091 too, showing the importance of residue F409. The hydrophobic contributions of the residues Y224, V296, L406, M407, F409, Y411, Y465 and Y514 are also common, other than M292 in case of P5091. The basic residues R408 and acidic residue D295 are also common between inhibitor 8WK and P5091 while K420 and D459 are additional in case of 8WK interaction. The aromatic box Y224, F409, Y465, H456 and Y514 is common between both molecules (Supplementary Figure 7). However, due to large size, 8WK has shown two additional features: 1) three residues of BL2 (D459, N460 and H461) are in vicinity, which we have not observed with P5091, and 2) other than two residues (D295 and V296) of SL which are common in both inhibitor, Q297 of same region has shown additional contribution to 8WK as it is forming H-bond with N-atom of 8WK. From the comparison it seems that P5091 possibly occupy the same site. Thus, overall mechanism of inhibition of P5091 at S7 proves our assumption of allosteric inhibition. The whole spectrum also explains the binding properties of P5091 by putting its binding impact on the key areas of USP7 and especially at the ubiquitin binding site highlighting itself as ubiquitin-competitive small molecule.

### *Hot-spots* mutation and its implication on net binding

Residues F409, Y465 and Y514 contributed significantly in the net binding energy. These residues also undergo significant conformational changes in different states. Therefore, we quantified the effect of these residues in net binding by inducing mutations. The combined mutants have shown remarkable drop of net binding free energy. However, individual mutants also have shown considerable drop in delta G (Supplementary Figure 8). These observations further confirm the criticality of the predicted *hotspot* residues essentially involved in the interaction with P5091.

## DISCUSSION

USP7 is a critical component in Ubiquitin proteasome system and its role is well established in tumorigenesis and cancer progression, makes it a target of immense interest [2, 3]. Many studies have revealed that selective inhibition of USP7 can be an efficient mechanism to arrest cancer [3]. The USP7 inhibitor destabilizes/decreases its substrate level including the MDM2 and increasing the level of P53 in various cancer [19–22]. Blocking USP7 facilitates the E3 ligase MdM2 degradation, and by doing so the USP7 inhibitor controls the tumor growth significantly [19]. Being pharmaceutically relevant target there are limited USP7 inhibitors reported so far, except few recently published active inhibitors [19–20]. The major bottleneck is the limitations of structural and dynamical information of possible allosteric binding sites formed between active/inactive states of USP7. Indeed, there are various crystal information available, though the thorough dynamic study has not been done to identify the possible binding sites on USP7 surface which can facilitate the rational of structure/ligand guided inhibitor designing. We explored the possible allosteric sites at USP7 in a ligand-dependent and independent manner, and for that inhibitor P5091 was used as chemical probe. P5091 is established in restricting multiple myeloma and few other cancers at *in-vitro* and *in-vivo* level [17, 18, 23, 24]. However, the binding site information and structural insights of P5091 is not yet reported. Other than enlightening the USP7-P5091 structural aspects of binding site and binding mode, we also performed a possible expansion of this study (i) for highlighting the conformational changes of USP7, (ii) the existence of multiple binding site, (iii) the druggability of identified binding site and, (iv) the possible inhibition mechanisms associated with identified sites. The notion is further supported by extensive implementation of computational methods to identify and characterize the potential binding site on USP7 by leveraging the information of known inhibitors.

From the site identification analysis, total eight sites were identified (from S0 to S7). From both the programs, a consensus results were obtained. The five main sites (S0, S1, S4, S6, and S7) were selected for detailed analysis. Apart from methodological cutoffs, the each predicted sites were scrutinized for its possible association with biological function.

From the MD simulations, we found S0 and S1are shallow pockets in nature as P5091 was unable to form stable interactions with their respective cavity-lining residues and it moved out from the pockets. A contrasting result was observed at site S6 where MD data fail to justify the blind docking and SiteMap results. Having highest docking energy and druggability score of P5091 at S6, it moved out from this site with high RMSD deviations. The exit from S6 is due to loss of pocket stability which is mainly due to the high flexibility of CH. This result reflects that only static analysis through docking is not enough for binding site prediction. Our result shows that lower half of CH is highly flexible which questions the binding of P5091 as it is not being able to stabilize CH and eventually moved out as the magnitude of fluctuation increases with trajectory (Supplementary Figure 2). Since this site claimed good druggability features (Dscore 0.98) at static states therefore, it provides a possibility for structure guided inhibitor designing to freeze the movement of CH by attacking charged patches (basic set of Lys residues), essential for USP7 activity [26]. Finally, two sites S4 and S7 were picked as most prominent and druggable binding site. The binding affinity is more favorable for S7 than S4, though S4 was picked for detailed quantitative analysis as its residue wise constitution resembles with S7 architecture. Both the sites have common amino acids like methionine (1/1), valine (1/1), phenylalanine (2/1), tyrosine (3/3) etc. The binding at S7 is mainly driven by non-polar forces as observed from high van der Waals contributions. The aromatic cage of residues Y224, F409, H456 and Y465 within 3.5 Å of dichlorophenyl (R2) ring of P5091 decreases the overall non-polar solvation penalty (Figure 3G and Supplementary Figure 4). This was holding true in the docking (cluster of 86 poses with docking score of −7.92) and the SiteMap (best druggability score of 0.87 and 0.99 for both M1 and M2, respectively).

Additionally, we also performed docking with some of the reported analogues of P5091. We found all the analogues of P5091 were preferably docked at S7 site with better docking energy and larger cluster size of conformations (Supplementary Figure 9). Moreover, study of binding pocket through electrostatic potential calculation and water dynamics helped us categorizing the favorable sites. From all the quantitative and qualitative results claimed S7 as a most promising site that possibly curb USP7 activity allosterically. The perturbed S7 in COM, hindered the important regions of USP7 especially, BL2 and S7 binding site area is mostly affected (Supplementary Figure 5). Comparison of S7 site was done with recently published most potent USP7 inhibitors [19–22]. We identified a tunnel which seems very critical for modulating the USP7 activity, as most of the inhibitors are in nano-molar range and they dynamically perturbed its key regions by binding at tunnel (Supplementary Figure 10). A huge conformational change was observed in the orientation of three key residues F409, Y465 and Y514 in multiple states of USP7 (APO, APO’ and COM). The significant conformational change at S7 site of APO (unbounded) state is providing the space for ligands to bind and settle down, which reflects incase of recently published USP7 inhibitors [22]. This space is not available in Ubiquitin bounded state. Also, the binding site volume seems to shrink remarkably in COM state in comparison with APO, explaining the tight packing of ligand at S7 due to significant conformational changes (Figure 6C). Targeting this site possibly hinder the ubiquitin positioning and restrict its C-terminal tail, essential for inactive to active state transition of USP7. Hence, binding of small molecule at S7 appears to be an ubiquitin-competitive small molecule.

## MATERIALS AND METHODS

### Selection of structures

To explore the most likely binding site of P5091, various available crystal structures of CD, CD+CH+UBL and CD+TRAF domains of USP7 were used. The initial data from blind docking suggests that good number of conformations and poses with better docking energy are achieved at CD and CD+CH+UBL. Henceforth, these two structures are used for further analysis.

### Protein structure preparations

Crystals 4M5W (APO) [25] and 5FWI (APO) [26] were obtained from Protein Data Bank The missing residues in 4M5W (L505-R508) and 5FWI (D459-N460 (BL2) and D502-H509) were interpolated by using PLOP algorithm [37] implemented in Schrodinger suite and the optimum conformation of loop was chosen on the basis of lowest energy. The loop conformations were optimized and then both the structures were minimized. An additional crystal 5JTJ (APO’) [38] which is only USP7-ubiquitin bounded protein was taken to observe the conformational changes of gatekeeper residues.

### P5091 preparation

The bioactive molecule P5091 selective inhibitor was taken from SELLEKCHEM database (http://www.selleckchem.com/). The molecule was prepared using Schrödinger's (version 2017-1) [39] LIGPREP [40], which generates tautomer’s, and possible ionization states at the pH range 7 ± 2 using Epik [41] and also generates all the stereoisomers of the compound if necessary. The optimization was done using the OPLS3 force field [42].

### Druggability testing

#### SiteMap analysis

SiteMap [43] program of Schrodinger Suite was also used for calculating binding site on two different structures: M1 and M2 (path A1 of Scheme 1). It identifies putative binding sites by implementing different parameters, which contributes to tight binding of P5091 with receptor. The different parameters on the basis of which a potential binding site is considered are: *site score, size, exposure score, enclosure, hydrophobic/hydrophilic character, contact, and donor/acceptor character*. As per Halgren [40] analysis, the average number of site for sub-micromolar sites is 132, where lower exposure scores of 0.52 and Higher exposure scores of 0.76 average is considered better for sub-micromolar sites. For the donor/ acceptor character and site score, the average for the sub-micromolar sites is 0.76 and 1.01, respectively. Druggability of site is denoted by Dscore. Dscore values provide a rough estimate of whether the site is druggable. These scores were derived by Halgren [44] by executing the SiteMap program on a number of proteins that have inhibitors bound with potencies in the sub-micromolar range and performing statistical analyses to produce optimized scores [44]. The OPLS-2005 force field [42] was employed, and a standard grid was used with 15 site points per reported site and cropped at 4.0 Å from the nearest site point.

### Molecular docking

To assess the robustness of results provided by above noted methods, we performed blind docking calculations (path A2 of Scheme 1). The docking studies were performed using AUTODOCK4.2 [45]. The docking in AUTODOCK was performed using two different protocols 1) Blind Docking [46] to explore the active sites in USP7 and to observe the P5091 favoring sites whether it is catalytic site or any other biologically relevant allosteric sites and 2) Focused Docking (path A3 of Scheme 1) to confirm the presence of clusters at active site by entering the docking grid on the center of mass of each site detected. The GLIDE version 7.4 [47] is also used for focused docking.

### Molecular dynamics simulation

MD simulations were carried out in DESMOND [48] module of Schrodinger Suite using the OPLS3 force field [42]. This study implements dynamics study of two APO proteins and six molecular systems of protein-P5091 complexes. The details of all these systems are described in (Supplementary Table 4). To begin with, the simulation systems were build using OPLS3 force field for proteins and solvated with SPC water model for USP7 models. Orthorhombic box shape was chosen, as it suits best for the globular proteins, with the edge length of 10 Å ensuring the minimal distance between atoms of protein and edge of the box. Counter ions were added to neutralize the systems. For USP7-P5091 docked complexes, the above parameters were same and counter ions were added at least 20 Å from ligand P5091. All prepared systems relaxed before the actual simulation by a series of energy minimization and short MD simulations. There are mainly six relaxation steps in this process where minimization of solute restrained and without restraints are carried in first two steps. Step three through six are short MD simulations of 12 ps, 12ps, 12ps and 24ps each using NPT ensemble each using the NPT ensemble at 10, 10, 300, and 300 k, respectively. In between at Step 5 the pocket is solvated as well. Velocity resampling is carried in steps three to five, while at step six it is not done.

The NPT ensemble was employed for the simulations with Nose-Hover chain thermostat and the Martyna-Tobias-Klein barostat. RESPA integrator was used with a time step of 0.002 ps. For short-range Coulombic interactions, a 9 Å cut off radius was considered. Bonds to hydrogen were constrained using the M-SHAKE algorithm of DESMOND. The simulation was carried out for total 100 ns for each system and the coordinates were saved at intervals of 20 ps that are referred to as “frames” in this study.

The Simulation Event Analysis module in DESMOND was run to further analyze the simulation results. This module generates raw data for all the required analysis. The root mean square deviation (RMSD) was calculated for the Cα atoms using the starting structure as reference frame, where all frames were superimposed on reference frame. And in the similar manner root mean square fluctuation (RMSF) and radius of gyration (Rg) is calculated.

### Clustering

Clustering is used to derive a small subset of protein structures whose pockets nevertheless provide coverage of the significant regions of the pocket conformational distribution. In particular, the CLARA algorithm [49] implemented in the R package cluster is applied to group the USP7 structures according to their scores. Retaining only the representative protein structure from each cluster leaves a subset of protein conformations corresponding to a diverse selection of binding site.

### Electrostatic potential calculations

The calculations were done using the APBS [50] program in VMD [51] for final complexes achieved by dynamics at S4 and S7. The protonated pqr file for both proteins was generated using pdb2pqr [52] module and the iso contour value of (+5 kTe^−1^) and (−5 kTe^−1^) was taken for positive and negative potentials respectively to generate the iso-surface of the protein.

### Energetic analysis

Free energy was carried out using the MMPBSA.py python script of AMBER tools and AMBER16 [53]. For this, the frames were extracted from the most stable state from the 100ns trajectory which was done using the VMD. So, overall 200 frames from all the protein-P5091 complexes were subjected to energy calculations. The binding free energy (*Δ*G_bind_) on each system is evaluated as follows:

*Δ*G_bind_ = G_com_−(G_rec_+G_lig_) (1)

where G_com_, G_rec_ and G_lig_ are the absolute free energies of com plex, receptor and P5091 respectively, arranged over the equilibrium trajectory. According to MM/PBSA method, the free energy difference can be decomposed as *Δ*G = *Δ*E_MM_ + *Δ*G_solv_ – T*Δ*S_conf_, where *Δ*E_MM_ is the difference in molecular mechanics energy, *Δ*Gs_olv_ the solvation energy (including an entropic contribution), and T*Δ*S_conf_ the solute configurational entropy (including the loss of translational and rotational entropy due to binding, as well as change in the vibrational entropy). The first two terms are calculated with the following equations:

Δ*E*_MM_ = Δ*E*_bond_ + Δ*E*_angle_ + Δ*E*_torsion_ + Δ*E*_vdw_ + Δ*E*_elect_ (2)

Δ*G*_solv_ = Δ*G*_PB_ + Δ*G*_SA_ (3)

where E_MM_ includes the molecular mechanics energy contributed by bonded (E_bond_, E_angle_, and E_torsion_) and nonbonded (E_vdw_ and E_elect_) terms of the force field and ΔGsolv is the solvation free energy, which has an electrostatic (ΔG_PB_, evaluated using the Poisson−Boltzmann equation) and a nonpolar contribution (ΔG_SA_ = γΔSA + β) proportional to the surface area (ΔSA).

Later, for estimation of the key residues which provides essential contribution to the stability of the P5091, the decomposition method implemented in AMBER16 was applied within the framework of the molecular mechanics (MM) Generalized Born Surface Area (GBSA) approach [54]. In addition to being faster than the MM-PBSA [55] approach, MM-GBSA [54] methods furnish an intrinsically easy way of decomposing the free energy of binding into contributions from single atoms and residues. These methods are implemented in sander module of AMBER16. The MMPBSA script was also used for calculating net binding energy after performing *in-silico* alanine scanning mutagenesis for *hot-spots* residues. Four different mutant's models were generated: M1 (F409A), M2 (Y465A), M3 (Y514A) and M4 (F409A+Y465A+Y514A) using MAESTRO.

### Watermap calculations

The calculations were done using WaterMap module [56] of Maestro. Four input protein structure were used for this calculation considering APO and COM of S4/S7 sites where binding site residues were used as parameters of their respective sites. The structures were prepared using the Protein Preparation Wizard in the Maestro. Amino acid residues outside of a 20 Å shell around P5091 were removed and the system was solvated in a TIP3P water box extending at least 10.0 Å beyond the truncated protein in all directions. A 2.0 ns MD simulation was performed following a standard WaterMap relaxation protocol with 5.0 kcal/mol positional constraints. Water molecules from the frames saved at 2.0 ps intervals in the simulation were clustered into distinct hydration sites, and the excess entropy and enthalpy were calculated relative to bulk solvent according to the inhomogeneous solvation theory [57].

### Cavity volume calculations

Additionally, pocket volume analysis was applied on site S7 to compare the volume of pocket in APO and COM. For this, we used POVME2 (Pocket Volume Measurer) algorithm [58]. We applied this algorithm on both APO and COM systems (S7). The trajectory was aligned and the frames were extracted from VMD [51] of both the systems, which is used as initial input for this method. In the next step, an inclusion and exclusion regions were defined where inclusion area encompasses all the binding pocket conformations of the trajectory while exclusion region is the area that does not associates with the pocket. For building sphere on which the inclusion grid-points are calculated, we chose C-atom of F409 that lies at the center of a cavity and also protruding inwards to it.

### Phylogeny and conservation analysis of possible binding pockets

Full length sequences of all Human-USPs were extracted from UNIPROT [59] and thereafter phylogenetic analysis was performed on the MUSCLE [60] generated alignment. The multiple -alignment was used for tree construction using the Neighbor-Joining algorithm [61]. The circular tree was constructed using the iTOL [62] utility by providing raw data in Newick-tree format. The leaves were colored on the basis of similarity within USPs. From the tree we chose five USPs (USP1, USP18, USP40, USP47) that are showing high similarity with USP7. The sequence of these USPs was taken into Multiple Sequence Viewer module of Maestro for finding the conserved residues at probable binding pockets.

### Figures

All the images were generated using VMD [51] and graphs were plotted using GRACE [63].

## CONCLUSIONS

We identified the possible druggable binding sites on USP7 and characterized them in ligand-dependent and independent ways as how these sites are critical for the modulation of USP7's biological activity. The dynamic nature of the identified two most prominent binding sites with the atomistic information elucidated through MD simulations provide meaningful molecular level insights for rationally structure-guided inhibitor designing with reduce unfavorable contacts and increased affinity.

## SUPPLEMENTARY MATERIALS FIGURES AND TABLES


