# Supplementary material for: Molecular dynamics simulation reveals the possible druggable hot-spots of USP7
Source: Oncotarget. 2018 Sep 28;9(76):34289–305. doi: 10.18632/oncotarget.26136 (PMC6188144; doi:10.18632/oncotarget.26136)
Supplement: Supplementary file 1 [file oncotarget-09-34289-s001.pdf]

Molecular dynamics simulation reveals the possible druggable *hotspots* of USP7

SUPPLEMENTARY MATERIALS

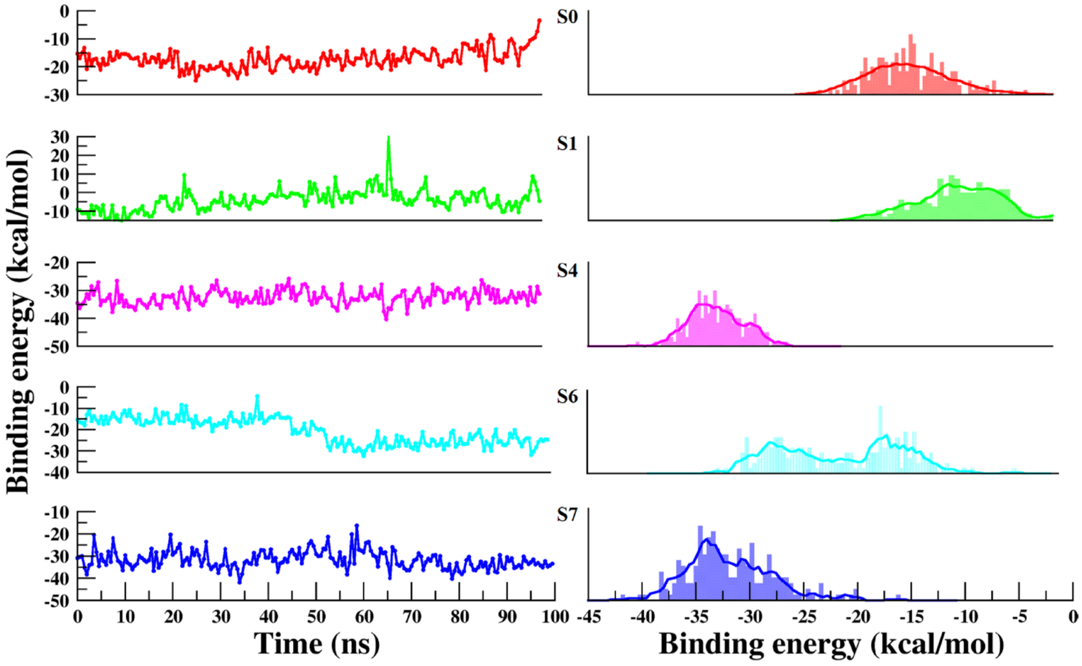

Supplementary Figure 1: The time evolution of binding free energy (MM/PBSA) throughout 100 ns at all different sites from S0 to S7.

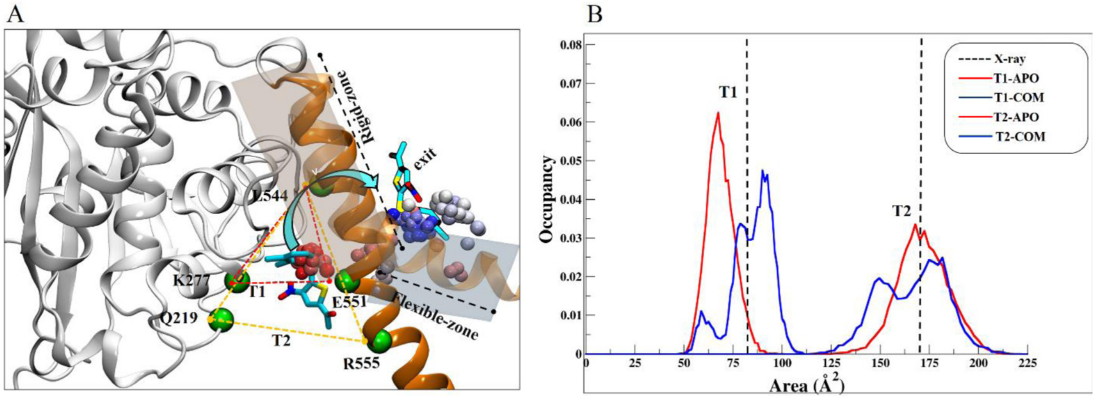

Supplementary Figure 2: Dynamics analysis on S6 pocket. (**A**) Shows the exit of molecule of P5091 and flexibility of connector helix (CH) in spite of high docking energy. P5091 is displayed is Cyan-ball and stick representation. CH- Orange. Beads correspond to the Cα atoms of residues (Q219, K277 (catalytic domain)- L544, E551 and R555 (CH)). (**B**) Distribution of the area defined by Cα atoms of residues K277-L544-E551 (T1) shown by APO (Blue) and complex (Red) and residues Q219-L544-R555 (T2) shown by APO (Red) and complex (Blue). The black vertical dotted lines indicate the value extracted from the X-ray structure.

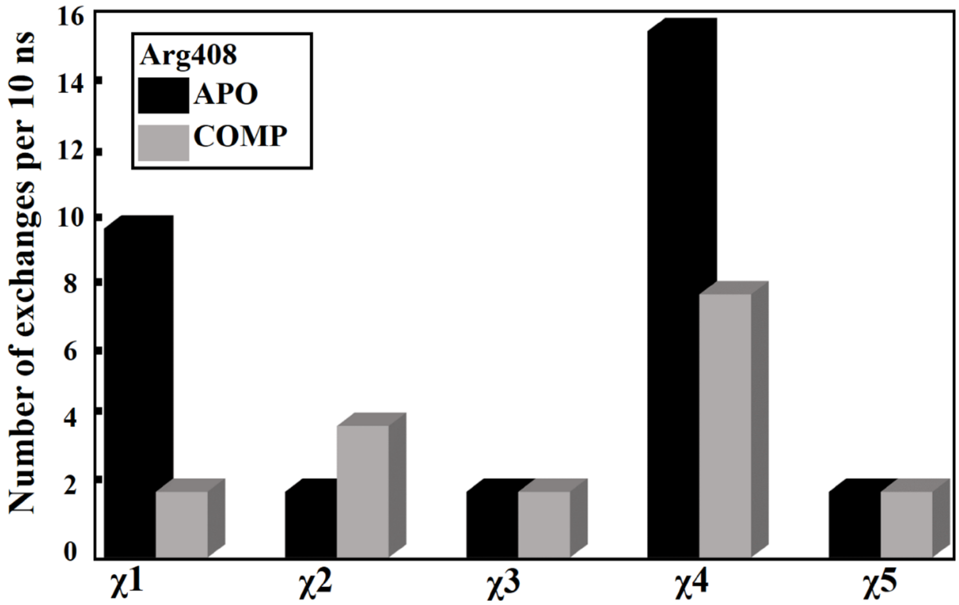

Supplementary Figure 3: Changes in the distribution of rotameric states of R408. The five rotameric states taken are chi1, chi2, chi3, chi4 and chi5.

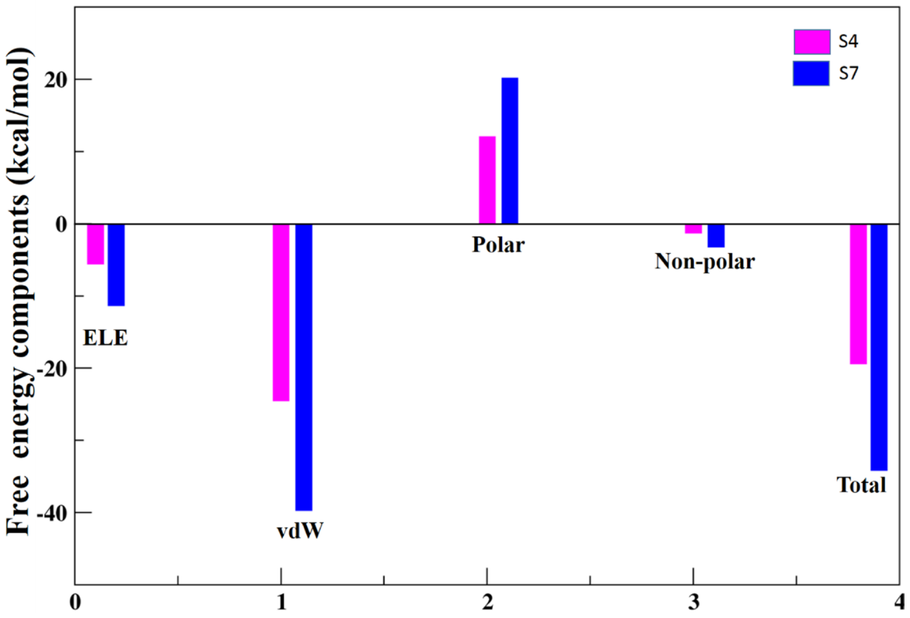

Supplementary Figure 4: Contribution of the binding free energy (in kcal/mol) components of P5091 at site S4 and S7.

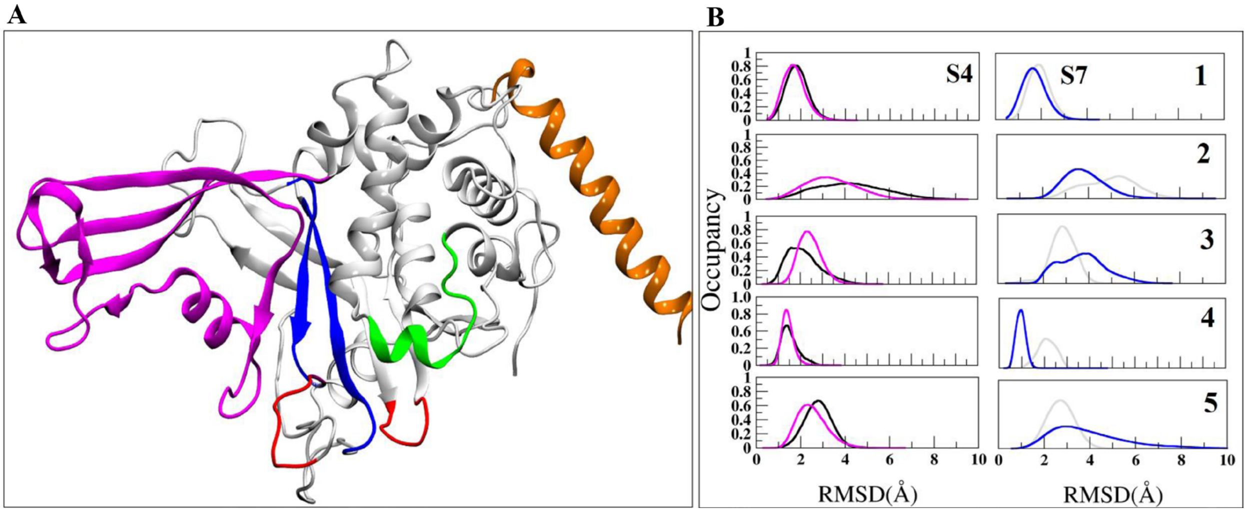

Supplementary Figure 5: Conformational analysis on potential perturbed areas in USP7 in systems APO-vs.-COM (S4 and S7). Selected areas highlighted in (**A**) and their respective deviations is shown in (**B**) as (1) Switching Loop (Green) (2) Blocking Loop1 (Red) (3) Blocking Loop2 (Red) (4) S4-binding site area (Magenta) and Ubiquitin attachment site (Magenta) (5) S7-binding site area (Blue). Panel 1-Black and Grey (APO) and Magenta and Blue (COM).

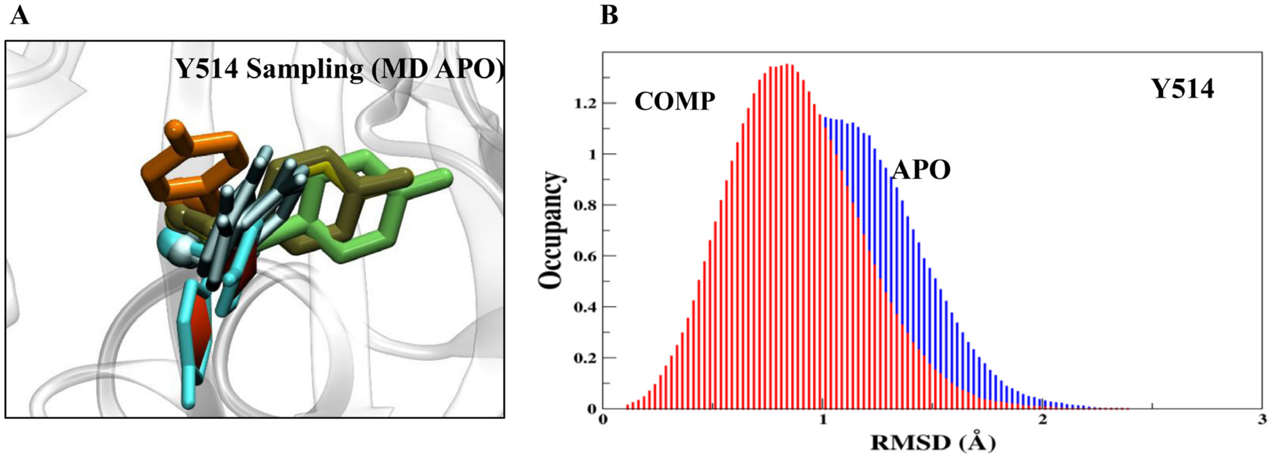

Supplementary Figure 6: Conformational sampling of Y514 in MD-APO. (**A**) Y514 sampling in MD-APO observed after superimposition of APO (lime), APO' (orange), MD-COM (yellow) and X-Ray-5NGE (tan). Extreme orientations of Y514 are shown in Cyan and the transient states of Y514 are shown in faded Cyan shows different states of Y514 in MD-APO. (**B**) the distribution of Y514 suggests that most of the states resembles to COM while a slight difference is observed.

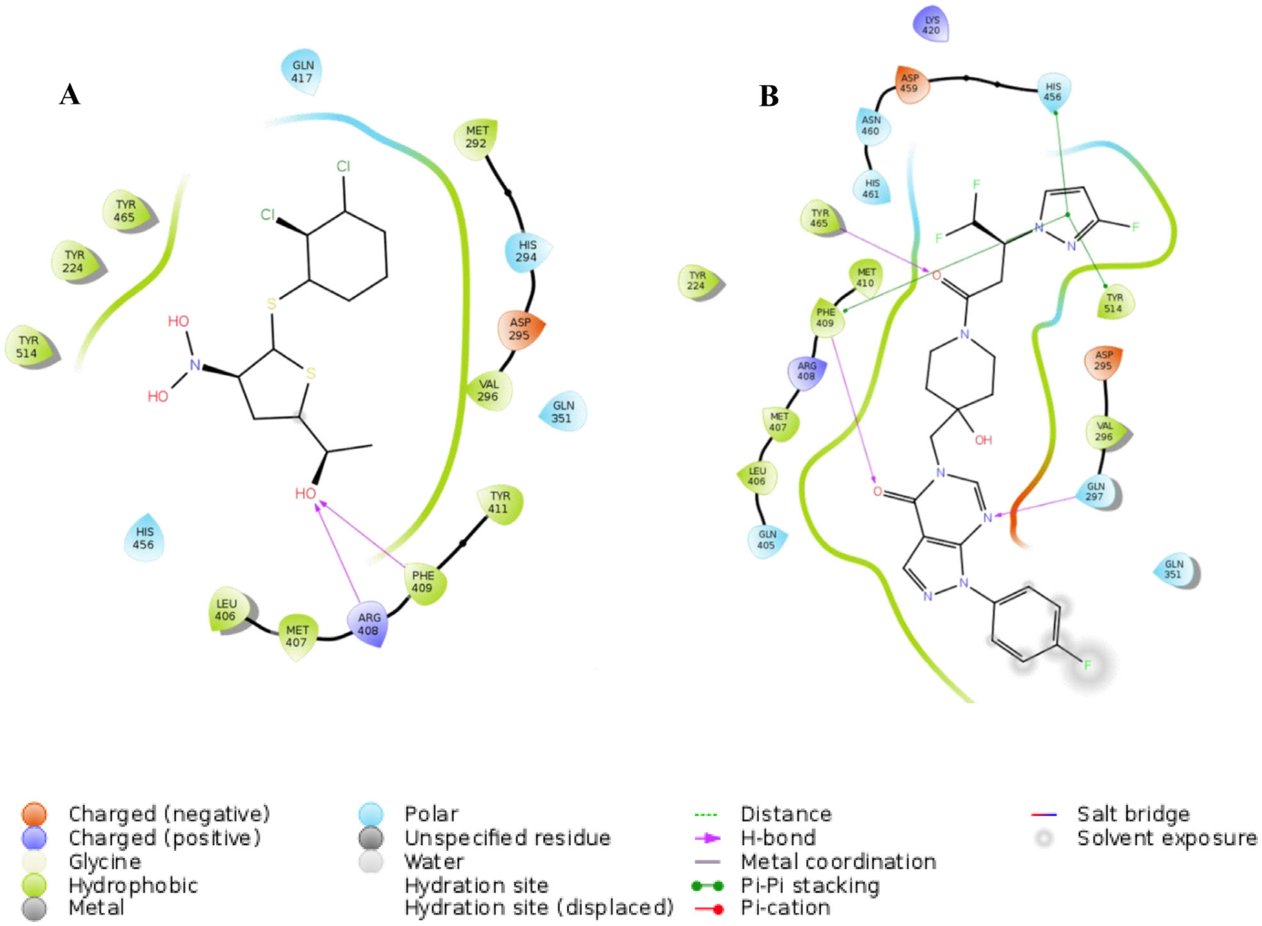

Supplementary Figure 7: Schematic of P5091 and 8WK/FT671 interaction at respective pockets. (**A**, **B**) residues lining cavity under 3.5 Å are shown with labelled hydrogen bonding and residues are displayed as per their chemical properties. The image was generated using Ligand Interaction Diagram of Maestro.

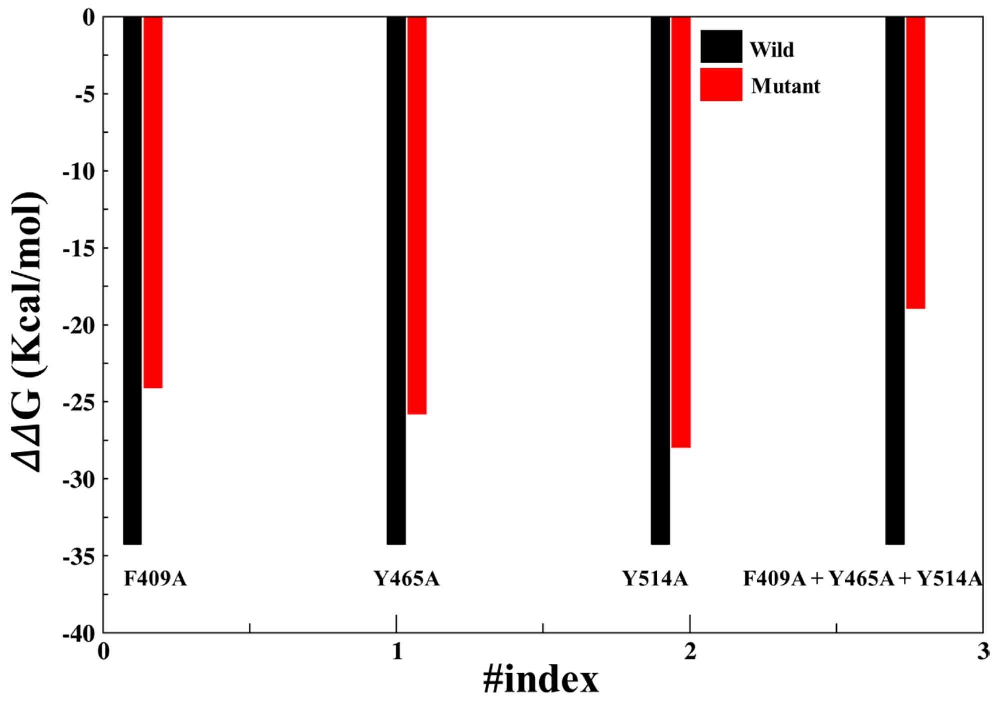

Supplementary Figure 8: The change in free binding energy of complex after mutating *hot-spot* residues with Alanine.

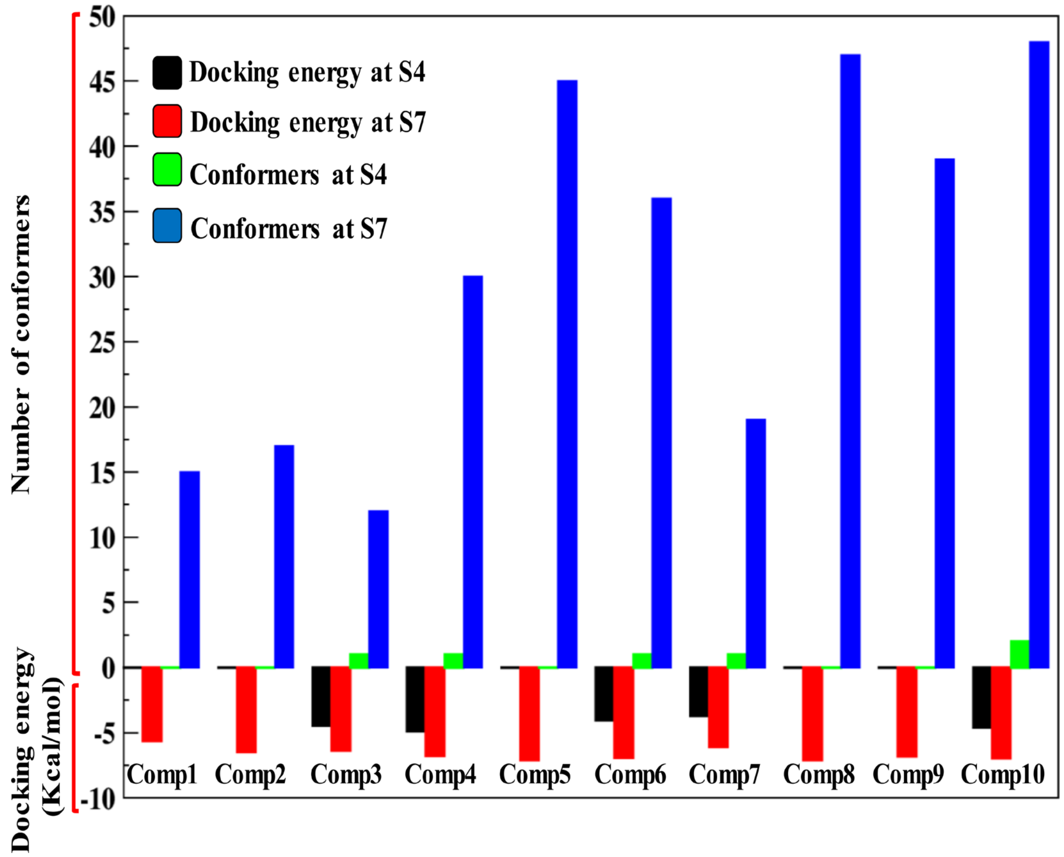

Supplementary Figure 9: Blind docking on P5091 analogues.

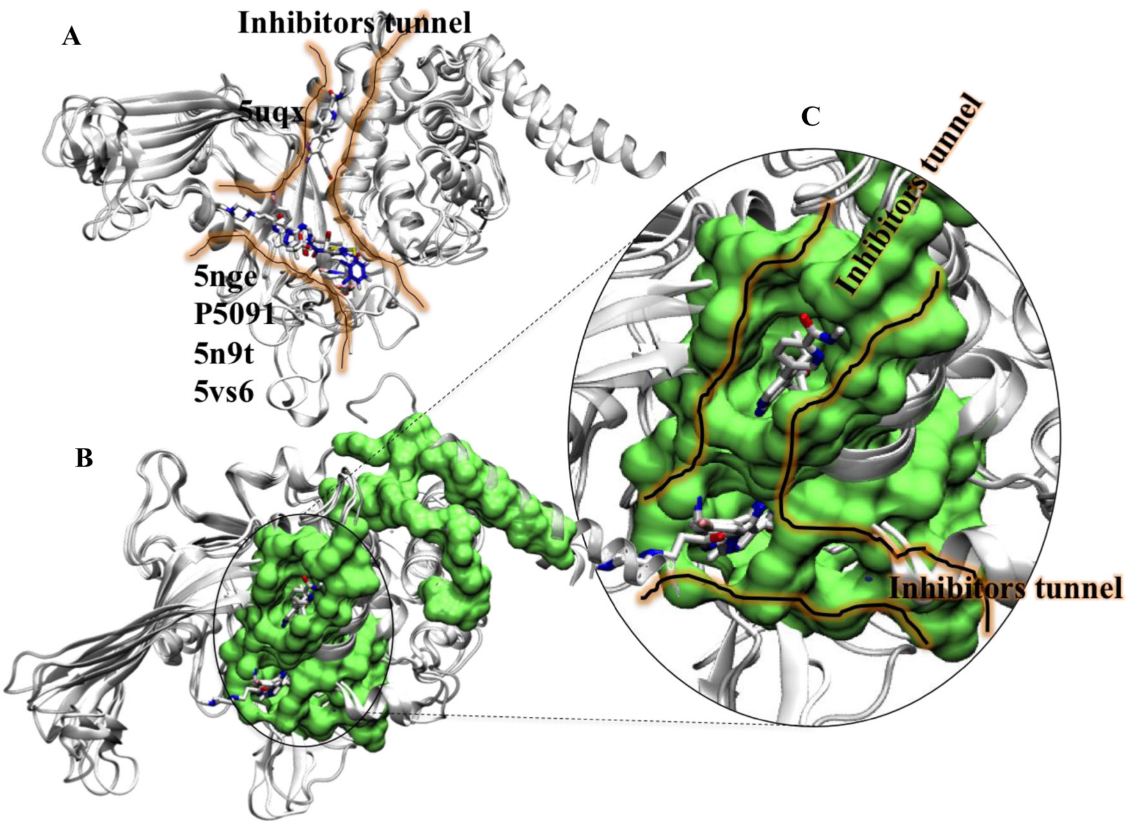

Supplementary Figure 10: Identified tunnel near site S7. P5091 and other recently reported inhibitors occupy this tunnel and modulate the activity of USP7. (**A**) Superimposition of Co-crystal with Comp-P5091, (**B**) Surface view of cavity (green) highlights the tunnel identified and (**C**) close-up view of tunnel.

Supplementary Table 1: SiteMap analysis on model M1

Site	SiteScore	size	Dscore	volume	exposure	Enclosure	contact	phobic	philic	balance	don/acc
S7	0.873037	66	0.866842	152.978	0.627119	0.686797	0.901003	0.415014	0.947532	0.437995	1.208445
S1	0.782166	59	0.773321	156.065	0.755187	0.598532	0.731033	0.399006	0.944251	0.422563	0.776332
S6	0.775734	65	0.689065	138.915	0.70852	0.575099	0.821102	0.067412	1.269469	0.053103	0.677904
S2	0.537548	19	0.324961	66.885	0.703125	0.624959	0.900498	0	1.410278	0	0.750536
S4	0.529218	22	0.429765	82.32	0.810345	0.576158	0.682671	0.043512	1.094565	0.039753	3.178519
S5	0.526255	20	0.448602	70.315	0.809524	0.576217	0.754351	0.259132	0.973952	0.266062	1.406307

Supplementary Table 2: SiteMap analysis on model M2

Site	SiteScore	size	Dscore	volume	exposure	enclosure	contact	phobic	philic	balance	don/acc
S6	1.047818	105	0.98446	279.202	0.435484	0.7695	1.005355	0.854019	1.279876	0.667267	0.393175
S7	1.008271	176	0.988654	325.507	0.48538	0.710366	0.935828	0.211236	1.158176	0.182387	1.747891
S1	0.971436	156	0.956844	436.639	0.597938	0.655286	0.872769	0.162497	1.154379	0.140766	0.891979
S2	0.786294	59	0.693933	174.244	0.681081	0.632672	0.774614	0.089752	1.250608	0.071767	1.305647
S0	0.599078	21	0.592257	60.025	0.827869	0.532569	0.553934	0.506945	0.484805	1.045668	0.926849

Supplementary Table 3: Binding site residues lining the cavities (S0, S1, S4, S6 and S7)

S0	S1	S4	S6	S7
N218	V213	M328	K217	Y224
G220	F233	Y347	G275	M292
C223	F234	D349	K277	H294
Q293	N236	Q351	E551	V296
G463	R239	L352	R555	Q351
H464	L267	F364	R558	L406
D481	Q268	Y367		M407
	P471	V393		R408
	K472	K394		F409
		F395		Y411
		F436		H456
				Y465
				Y514

The residues at respective sites were taken at 3.5 Å cut from P5091 docked at different sites. Furthermore, they were used to plot conservation amongst chosen USPs (USP7, USP1, USP18, USP40, USP47).

Supplementary Table 4: Total number of systems subjected to MD simulations (each 100ns)

ID	Systems	Time (ns)	Total no. of atoms	Water molecules	Ions
1	USP7-CD-Crystal	100	45211	39752	8 Na^+
2	USP7-CD-Model	100	45377	39651	8 Na^+
3	USP7-CD-CH-Crystal	100	53856	48389	12 Na^+
4	USP7-CD-CH-Model	100	53979	48273	12 Na^+
5	USP7-CD-CH – 'S0'	100	44797	39831	8 Na^+
6	USP7-CD-CH – 'S1'	100	44696	39684	7 Na^+
7	USP7-CD-CH – 'S4'	100	44833	39867	7 Na^+
8	USP7-CD-CH – 'S6'	100	53985	48279	12 Na^+
9	USP7-CD-CH – 'S7'	100	51169	45361	12 Na^+
